# Compatibility of intravenous ibuprofen with lipids and parenteral nutrition, for use as a continuous infusion

**DOI:** 10.1371/journal.pone.0190577

**Published:** 2018-01-03

**Authors:** Jowell Garcia, Alka Garg, Yunmei Song, Ambados Fotios, Chad Andersen, Sanjay Garg

**Affiliations:** 1 School of Pharmacy and Medical Sciences, University of South Australia, Adelaide, South Australia, Australia; 2 SA Pharmacy, Women's & Children's Hospital, Women’s and Children’s Hospital, North Adelaide, South Australia, Australia; 3 Department of Neonatal Medicine, Women’s and Children’s Hospital, North Adelaide, South Australia, Australia; Izmir Katip Celebi Universitesi Tip Fakultesi, TURKEY

## Abstract

There is increasing interest to administer ibuprofen as a continuous infusion instead of a traditional bolus for treating Patent Ductus Arteriosus (PDA). However, its compatibility data with commonly used drugs in the neonatal period, including parenteral nutrition (PN) and lipids is unavailable. The aim is to determine the compatibility of intravenous ibuprofen lysine with various ANZNN parenteral nutrition consensus group standard neonatal PN formulations and lipids. The PN and lipid solutions used in a tertiary neonatal unit were obtained. These included a Starter, Standard Preterm and low carbohydrate PN, and IV SMOF lipid admixture (SMOFLipid 20% 15 mL; Vitalipid N infant 4 mL, Soluvit N 1 mL) plus vitamin mixtures. 10% glucose was used as a control. 1:1 mixtures of different concentrations (1.25 to 5mg/mL) of ibuprofen lysine and each of the PN/glucose/lipid formulations were made. Samples were taken at hourly intervals for a total of 4 hours and tested for both physical (visual assessment, pH and microscopy) and chemical compatibility (High Performance Liquid Chromatography analysis). Zeta potential and particle diameter were measured for SMOF lipid admixture and ibuprofen combination to assess emulsion stability. 24 hour stability of ibuprofen dilution in 5 mL BD Luer-lok polypropylene syringes at 25°C was also assessed. Most PN formed opaque solutions when mixed with ibuprofen 2.5 and 5mg/mL solutions. However, ibuprofen dilution of 1.25mg/mL produced clear, colourless solutions with no microscopic particles when mixed with all PN/glucose/lipid formulations tested. Ibuprofen was chemically stable with all PN and SMOF lipid admixture, for a period of 4 hours. The zeta potential and particle diameter were within acceptable limits. Ibuprofen lysine was stable over 24 hours in Luer-lok polypropylene syringes. Ibuprofen 1.25mg/mL is physically and chemically compatible with 10% glucose, starter PN, standard preterm and low carbohydrate PN, and SMOF lipid admixture plus vitamins for a period of four hours, which is the maximum time they could be in an admixture during a continuous infusion.

## Introduction

Patent ductus arteriosus (PDA) affects up to 80% of infants weighing less than 1.2 kg [1]. It is characterised by an opening (patent) between the aorta and pulmonary artery called the ductus arteriosus (DA), which following delivery leads to left to right shunting of blood. The volume is dependent on the pressure difference between the systemic and pulmonary circulations and the DA size. The duct closes soon after birth in term newborn but is associated with serious morbidities in the preterm newborn including necrotising enterocolitis, bronchopulmonary dysplasia, respiratory distress and intraventricular haemorrhage. Pharmacological treatment consists of using non-steroidal anti-inflammatory drugs (NSAIDS), typically Indomethacin or Ibuprofen. There is an ongoing debate about which of the two NSAIDS is best suited for this intervention [2]. A Cochrane review published in 2015 suggested Ibuprofen to be as effective as indomethacin in closure efficacy but with reduced renal and gastric complications, thereby making it an agent of choice in many neonatal units [3]. Lately a few reports have also been published using oral or intravenous paracetamol with varying success [4–6].

A few recent publications have sparked interest in the use of Ibuprofen as a continuous infusion instead of the three daily bolus doses. The studies suggest that higher ductal closure rate might be achieved with a continuous dosing strategy and that a steady level of of ibuprofen may be more important for ductal closure [7].

The potential use of a continuous infusion of ibuprofen raises a question of the availability of dedicated lines for its administration. Neonates receiving this drug often also receive a number of other intravenous infusions including total parenteral nutrition (TPN) and lipid admixtures (LA). However, very limited data regarding the compatibility of ibuprofen with TPN and LA.

Holt et al. investigated the compatibility of ibuprofen lysine with TPN [8]. They analysed two parenteral nutrition products—TPN Electrolytes and Intralipid 10%. They reported that TPN Electrolytes was incompatible with ibuprofen lysine due to precipitate formation whereas Intralipid mixed with ibuprofen did not change in appearance, form precipitate or undergo phase separation. The authors concluded that their methods were insufficient in evaluating the compatibility of this combination.

The aim of this study was to determine the physical and chemical compatibility of intravenous ibuprofen with various commonly used neonatal TPN and LA, with a potential view of co-administering them through the same line via Y-site connectors.

## Materials and method

The study was conducted by Women’s and Children’s Hospital in Adelaide, South Australia (WCHN) in collaboration with the School of Pharmacy and Medical Sciences, University of South Australia. WCHN uses the ANZNN parenteral nutrition consensus neonatal TPN formulae. The TPN mixtures studied include Starter PN, Standard Preterm PN, Low carbohydrate Preterm PN. 10% glucose served as the control. The formulations of these PN are described in Table 1. IV SMOFLipid admixture (composition in Table 2) was the LA tested.

**Table 1:** Composition of parenteral nutrition tested (Source: Women and Children’s Hospital).

Composition of Parenteral Nutrition Tested			
Composition (per 100mL)	Starter PN	Standard preterm PN	Low carbohydrate preterm PN
Amino acid (as Primene)	3.3 g	3 g	3 g
Glucose	10 g	10 g	7.5 g
**Approx. electrolyte (mmol)**			
Sodium	1.5	3.3	3.3
Potassium	0	2.2	2.2
Chloride	0.93	1.35	1.35
Calcium	1.2	1.2	1.2
Magnesium	0.15	0.15	0.15
Phosphate	1	1	1
Acetate	0.5	4	4
Zinc	0 mg	0.326 mg	0.326 mg
Selenium	0 mcg	2 mcg	2 mcg
Iodide	0 mcg	0.8 mcg	0.8 mcg
Heparin	50 Units	50 Units	50 Units
Nitrogen content	0.495 g	0.45 g	0.45 g

**Table 2:** Composition of IV SMOFLipid admixture (Source: Women and Children’s Hospital).

IV SMOFLipid admixture Composition/20 mL Syringe					
SMOFLipid (15 mL)		Soluvit N Infant (1 mL)		Vitalipid N Infant (4 mL)	
**Refined soybean**	900 mg	**Thiamine nitrate**	3.1 mg	**Retinyl palmitate (Vitamin A)**	0.276 mg
**Medium chain triglycerides**	900 mg	**Sodium riboflavin phosphate**	4.9 mg	**Ergocalciferol (Vitamin D**_**2**_**)**	0.004 mg
**Refined olive oil**	750 mg	**Nicotinamide**	40 mg	**dl-α-tocopherol (Vitamin E)**	2.56 mg
**Glycerol**	375 mg	**Pyridoxine hydrochloride**	4.9 mg	**Phytomenadione (Vitamin K**_**1**_**)**	0.08 mg
**Purified egg phospholipids**	180 mg	**Sodium pantothenate**	16.5 mg	**Soya oil**	400 mg
**All-*rac*-α-tocopherol (Vitamin E)**	2.4–3.45 mg	**Sodium ascorbate**	113 mg	**Egg lecithin**	48 mg
**Sodium hydroxide**	To pH approx. 8	**Biotin**	0.06 mg	**Glycerol**	88 mg
**Sodium oleate**	4.5 mg	**Folic acid**	0.4 mg	**Sodium hydroxide**	To pH 8
**Water for injection**	To 15 mL	**Cyanocobalamin**	5 μg	**Water for injection**	To 4 mL
		**Glycine**	300 mg		
		**Edetate sodium**	0.5 mg		

**Fig 1:**
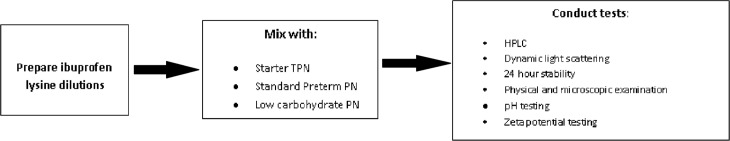
Methodology of study.

While various salts of Ibuprofen are currently available, Ibuprofen lysine was being used in our neonatal unit at the time of this study. Different ibuprofen concentrations ranging from 1.25 mg/mL to 5 mg/mL were tested (see Tables 1 and 2). The Methodology of this study is shown in Fig 1.

### Ibuprofen lysine

Neoprofen^tm^ was used in the study, available as 10 mg/mL ibuprofen solutions in 2 mL borosilicate glass vials. Three dilutions were tested, first for 24hr stability in solution and then for compatibility with TPN and LA—5 mg/mL, 2.5 mg/mL and 1.25 mg/mL Neoprofen^tm^ solutions were prepared in the laboratory by diluting it with 0.9% Sodium.

Stock ibuprofen lysine powder was purchased from BOSC Sciences. 17.1 mg of the powder was mixed with 2 mL RO water to produce a stock ibuprofen 5 mg/mL solution. The purity of this stock was 99% and confirmed by HPLC.

### Parenteral nutrition and lipid admixtures

Starter, Preterm Standard and low carbohydrate Preterm solutions (ANZNN parenteral nutrition consensus formulae) and 10% glucose bags were obtained from Baxter Pharmaceuticals. IV SMOFLipid admixture syringes (20mL in a clear BD 50mL amber polypropylene B&D syringe) were made in house under sterile conditions, by validated sterile trained staff. All formulations were stored in light protecting bags at 4°C.

### Analytical method development

Chemical compatibility was assessed using HPLC. The system consisted of a Shimadzu LC-20 AD Prominence Liquid Chromatogram connected to a DGU-20AS Prominence Degasser, SIL-20A HT Prominence Autosampler and SPD-M2DA Prominence diode array detector.

The HPLC protocol was adapted and modified from the British Pharmacopeia (BP) ibuprofen monograph. Two degassed mobile phases, A and B, were pumped through an Altima 4.6 mm x 250 mm C18 column at 1.5 mL/min for 22 minutes per sample. Mobile phase A consisted of 0.5% orthophosphoric acid, 34% acetonitrile and 66% RO water. Mobile phase B was pure acetonitrile. The detection wavelength and injection volume were set to 230 nm and 20 μL, respectively.

1:1 mixtures of ibuprofen lysine and PN/glucose/IV SMOFLipid admixture were mixed in 1.5 mL centrifuge tubes and left out at room temperature. At hourly intervals for a period of 4 hours, 80 μL aliquots were extracted and diluted to a concentration of 0.1 mg/mL. Supernatant from this solution was analysed by means of HPLC. Each time point was performed in triplicate. The mean concentration for each time point was compared against the initial ibuprofen concentration to determine the percentage recovery. Standard deviations and 95% confidence intervals were calculated using SPSS statistical software and the McCallum Layton Stats Calculator, respectively.

### HPLC method validation

The assay was validated by determining the linearity, precision and accuracy as per the ICH Guidelines. Freshly prepared stock ibuprofen 5 mg/mL solution was used for these experiments. Linearity was determined by producing two standard curves per day for 3 days with six concentrations and comparing the mean r^2^ and slope.

Accuracy was validated by preparing three separate stock solutions of 80 μg/mL (80%), 100 μg/mL (100%) and 120 μg/mL (120%). 200 μL aliquots were analysed with HPLC. The same aliquots were re-run 6 hours later. The mean percentage recovery and standard deviation and standard deviation were obtained for each concentration value.

Precision was validated by analysing one 100 μg/mL sample six times twice a day for three days. Validation of these 3 parameters was done by determining and comparing the intra-day and inter-day coefficient of variation. The detection and quantification limits were determined by determining the peak to height ratio of very low ibuprofen concentrations.

### 24 hour stability

1.25 mg/mL dilution of ibuprofen lysine was tested for 24 hr stability in solution. The final solution was left in the 5 mL syringe. The syringes were left in the laboratory and the temperature was monitored for up to 24 hours. Two 20 μL aliquots were removed from the syringe and diluted with RO water. Supernatant from the dilution was immediately subjected to HPLC analysis.

### Physiochemical compatibility assessment

#### Visual observation

Solutions of pure ibuprofen, PN and ibuprofen-PN admixture were assessed visually according to British Pharmacopeia method 2.2.1. 180 μL aliquots were taken out at 0, 30 minutes, 1 hour, 4 hours and 24 hours and placed into 200 μL glass HPLC tubes. Under white light and against a dark background, the tubes were analysed for signs of precipitation, gas formation and changes in colour. A laser light was shined through all mixtures for signs of the Tyndall Effect as this only occurs if particles were suspended in solution. Pictures were taken for further assessment.

IV SMOFLipid admixture and Ibuprofen mixtures were not tested visually due to the opaque milky white nature of the LA originally.

#### Microscopy

10 μL of mixture were microscopically examined for particles with an Olympus BX51 microscope. Samples containing particles were compared against calcium monohydrogen phosphate dihydrate crystals (Sigma-Aldritch), pure ibuprofen 5 mg/mL and pure PN.

#### pH measurements

Measurements from two of each—ibuprofen-TPN and ibuprofen-LA were taken at 0, 1, 2, 3 and 4 hours. An Orion PerpHecT Ross pH electrode (Thermo Scientific) was utilised to make the measurements. The pH results were put into the Henderson-Hasselbach equation to determine the percentage formation of monohydrogen phosphate ion (HPO_4_^2-^). This quantified the risk of calcium monohydrogen phosphate dihydrate (CaHPO_4_.H_2_O) precipitation.

#### Particle diameter determination and zeta potential

Particle size assessment via dynamic light scattering (DLS) and zeta potential measurement were employed to assess physical compatibility between LA and ibuprofen. A Zetasizer Nano ZS (Malvern Technologies) was utilised to conduct the DLS and zeta potential assessments. 5 μL samples were diluted with 1 mL of 0.2 μm–filtered RO water in a 40 μL cuvette. The intensity-weighted mean particle diameter and polydispersity index were recorded from the DLS as indicators of IV SMOFLipid admixture stability.

To determine the zeta potential, standard solutions of particles with zeta potentials of -60 mV and -45 mV were used for calibration. 5 μL of pure IV SMOFLipid admixture and SMOFLipid admixture -ibuprofen mixtures were diluted with 1 mL RO water. They were added into separate Zetasizer DTS1060 cells and run through the machine. The experiments were performed in duplicate.

### Chemical compatibility assessment

This was done using the HPLC method developed and validated in-house. IV SMOFLipid admixture, whether undiluted or mixed with another injection solution, is a milky, opaque emulsion containing thousands of lipid nanoparticles which needed to be removed. Mixing 20 μL of SMOF/ibuprofen with 480 μL methanol and 500 μL acetonitrile removed these particles and produced a clear, colourless solution. An aliquot was immediately analysed with HPLC. This method was validated by running IV SMOFLipid admixture /ibuprofen samples of known concentration and determining the percentage recovery from a standard curve. This method resulted in 100% recovery of ibuprofen.

1:1 mixtures of ibuprofen lysine and TPN/glucose/IV SMOF lipid admixture were mixed in 1.5 mL centrifuge tubes and left out at room temperature. At hourly intervals for a period of 4 hours, 80 μL aliquots were extracted and diluted to a concentration of 0.1 mg/mL. Supernatant from this solution was analysed by means of HPLC. Each time point was performed in triplicate. The mean concentration for each time point was compared against the initial ibuprofen concentration to determine the percentage recovery. Standard deviations and 95% confidence intervals were calculated using SPSS statistical software and the McCallum Layton Stats Calculator, respectively.

## Results

### HPLC validation

**Fig 2:**
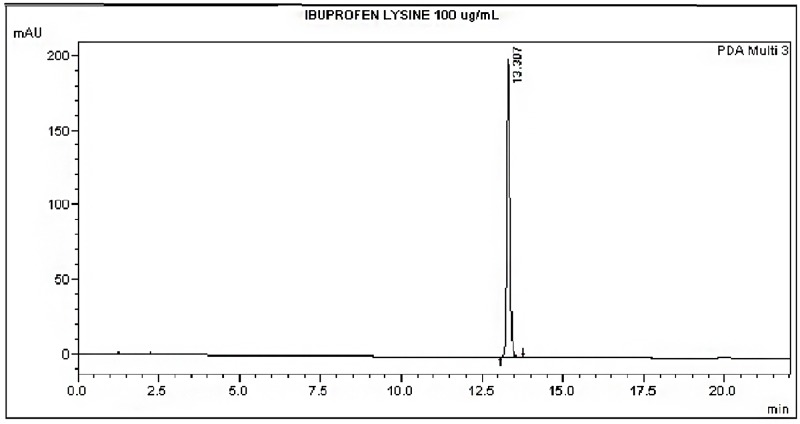
Chromatogram of ibuprofen lysine at time 0 hours.

**Fig 3:**
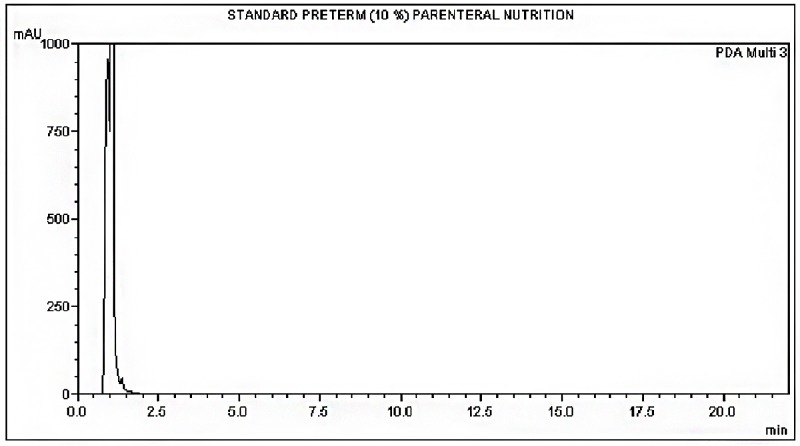
Chromatogram of standard preterm PN at time 0 hours.

**Fig 4:**
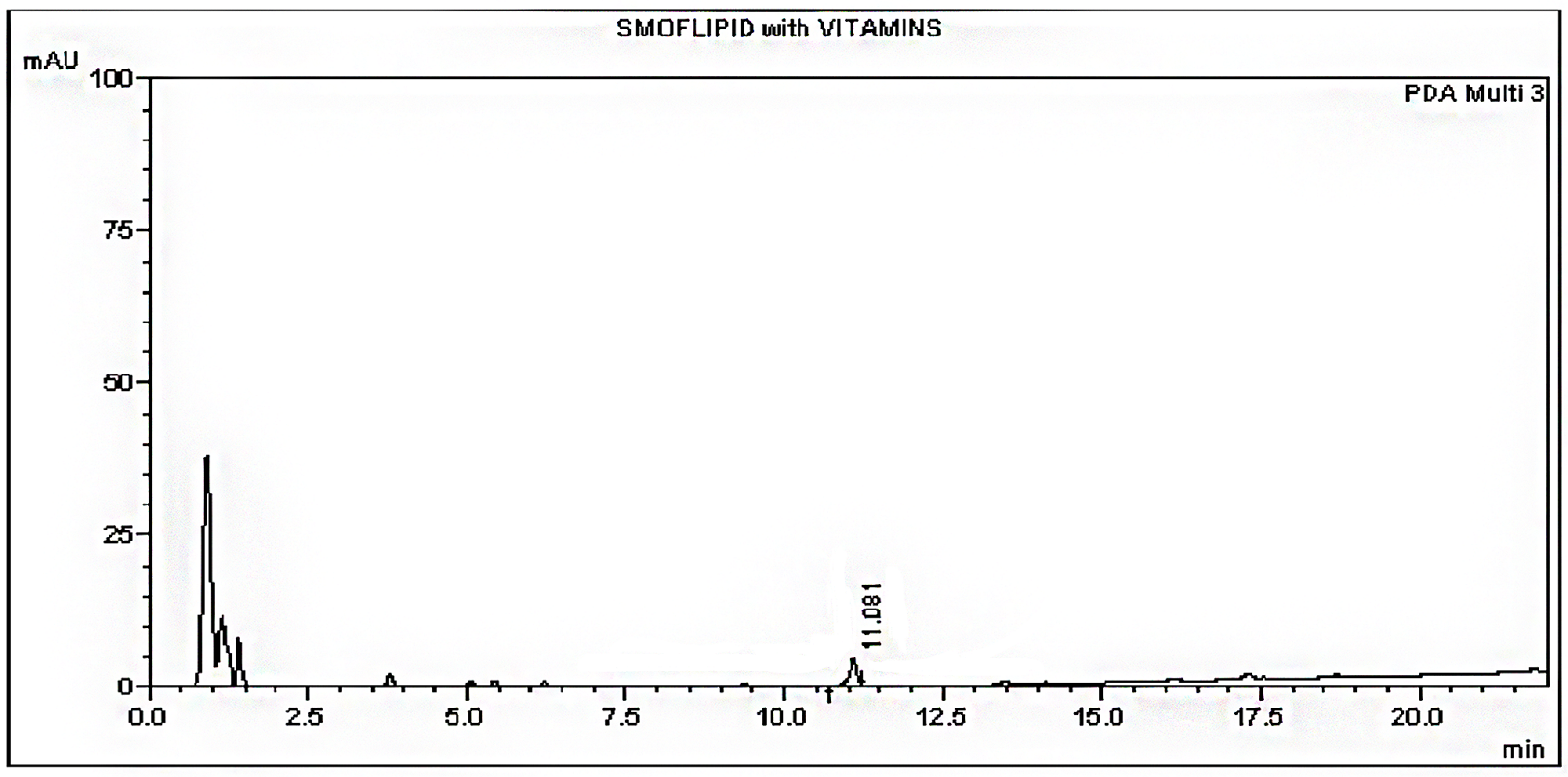
Chromatogram of IV SMOFLipid admixture + vitamins at time 0 hours.

The HPLC method was able to detect the ibuprofen lysine and there was no interference from the formulations (Figs 2, 3 and 4). A linear standard curve was produced. The average slope and R^2^ were 12177.3 and 0.999, respectively.

The HPLC method was shown to be precise and accurate when pure ibuprofen samples were tested. The intra-day and inter-day variability were below 5%, which is acceptable for industrial standards.

### 24 hour stability

At a temperature of 23 C^o^, the ibuprofen lysine did not degrade in the syringe during the 24 hour period (Table 3). There was no significant change of pH values.

**Table 3:** Ibuprofen stability data.

Ibuprofen 1.25 mg/mL 24 hour Stability		
	Time = 0 hours	Time = 24 hours
**Average ibuprofen concentration (**μ**g/mL) ± SD**	141.51	142.70 ± 12.6
**Average ibuprofen % recovery ± SD**	-	100.85 ± 12.9
**pH**	6.59	6.71

### Visual observation

**Fig 5:**
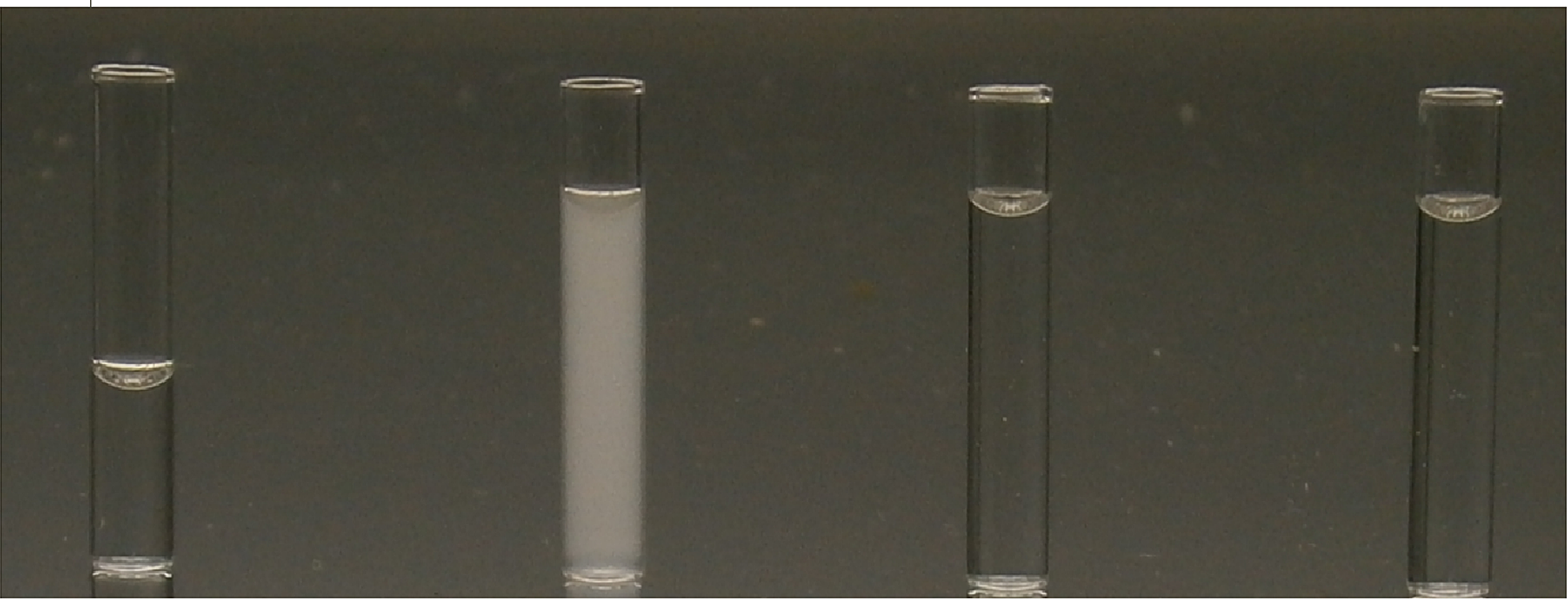
Visual observation at time = 0 hours. From left to right: Appearance of Ibuprofen 5 mg/mL diluted with 0.9% NaCl, ibuprofen 5 mg/mL mixed 1:1 with standard preterm PN 10%, ibuprofen 2.5 mg/mL mixed 1:1 with standard preterm PN 10% and ibuprofen 1.25 mg/mL mixed 1:1 with standard preterm PN 10%.

**Fig 6:**
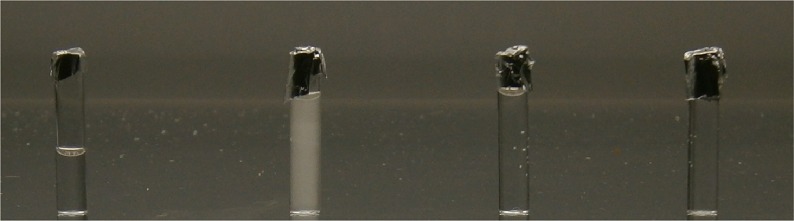
Visual observation at time = 4 hours. From left to right: Appearance of ibuprofen lysine 5 mg/mL diluted with 0.9% NaCl, ibuprofen 5 mg/mL mixed 1:1 with standard preterm PN 10%, ibuprofen 2.5 mg/mL mixed 1:1 with standard preterm PN 10% and ibuprofen 1.25 mg/mL mixed 1:1 with standard preterm PN 10%.

The following formulations formed opaque, hazy solutions when mixed with ibuprofen 5 mg/mL; starter TPN, low carbohydrate and standard preterm PN (Table 4; Fig 5). All solutions produced a Tyndall effect when the laser was shined through. The hazy, white layer was persistent for up to 4 hours (Fig 6). Sediment was detected after 24 hours. Mixing ibuprofen concentration 2.5 mg/mL with standard preterm 10% PN and 7.5% glucose PN produced clear, colourless solutions with no Tyndall effect. However, this did not occur with starter TPN until it was mixed with ibuprofen 1.25 mg/mL.

**Table 4:** Appearance of ibuprofen lysine mixed with PN/glucose/IV SMOFLipid admixture at time = 0 hours.

Visual Appearance of Ibuprofen + PN/Glucose/Lipid			
	Ibuprofen Lysine Concentration at Time = 0 hours		
Parenteral Nutrition Type	5 mg/mL	2.5 mg/mL	1.25 mg/mL
Starter TPN	Cloudy, opaque solution	Slightly cloudy, opaque solution	Clear, colourless solution
Standard Preterm 10% Neonatal PN	Cloudy, opaque solution	Clear, colourless solution	Clear, colourless solution
7.5% Dextrose Preterm PN	Cloudy, opaque solution	Clear, colourless solution	Clear, colourless solution
10% Glucose	Clear, colourless solution	Clear, colourless solution	Clear, colourless solution
SMOF lipid admixture + vitamins	Milky and turbid	Milky and turbid	Milky and turbid

10% glucose remained clear and colourless when mixed with ibuprofen of any concentration. No particles were observed under the microscope. IV SMOFLipid admixture combined with vitamins and ibuprofen 1.25 mg/mL showed no signs of cracking or phase separation over the 4 hour period.

### Microscopic observation

**Fig 7:**
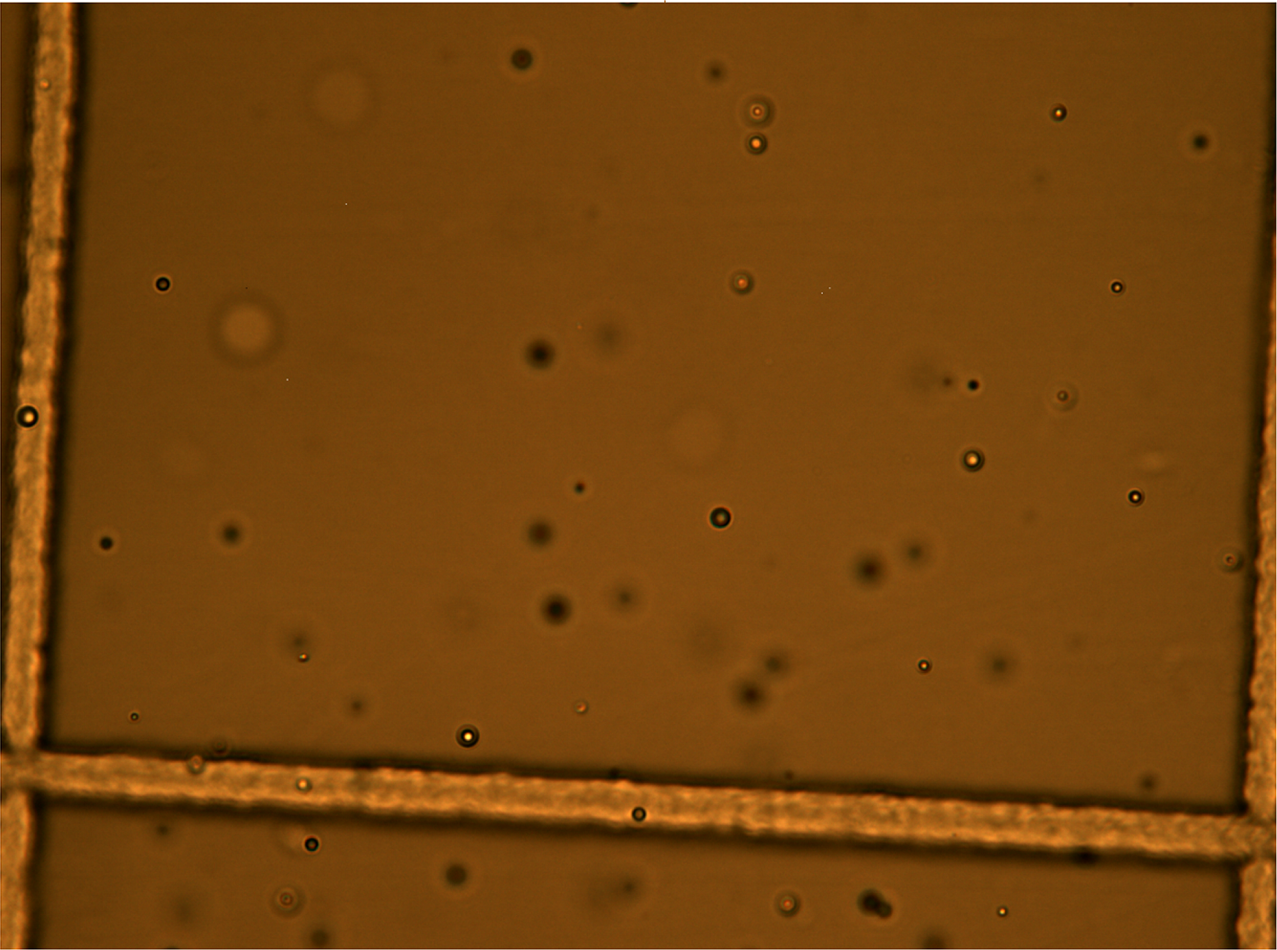
Ibuprofen 5 mg/mL + standard preterm PN.

The particles produced in the Starter, Standard Preterm and low carbohydrate PN mixed with ibuprofen were spherical, numerous and did not look like calcium phosphate crystals (Fig 7). These particles were present for up to 4 hours. Examination of pure ibuprofen lysine and PN (excluding IV SMOFLipid admixture) did not find these particles in solution. These particles were absent in ibuprofen 1.25 mg/mL mixed with PN.

### pH measurements

**Fig 8:**
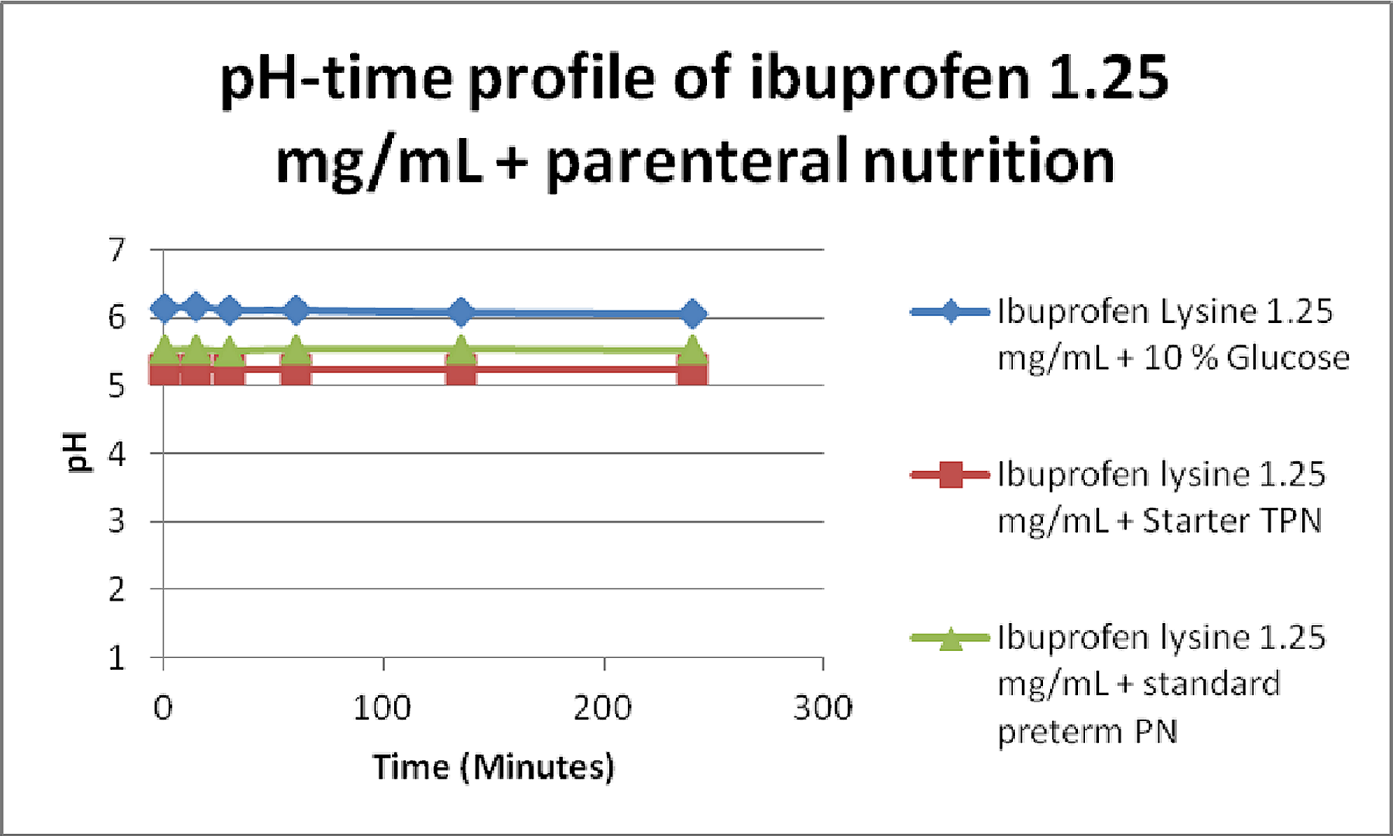
Change in pH over 4 hours in ibuprofen + PN/glucose combinations.

The pH of all 3 PN was between 5.2 and 5.5. Addition of ibuprofen lysine 1.25 mg/mL to 5 mg/mL to the 3 PN formulations did not increase the pH significantly (Fig 8). The percentage formation of HPO_4_^2-^ increased but was negligible and insignificant. Along with visual inspection and microscopy results, it is proposed CaHPO_4_.H_2_O crystals did not form in the three incompatible PN mixtures.

The pH of pure IV SMOFLipid admixture and IV SMOFLipid admixture mixed with ibuprofen was initially 6.9 and 7.4, respectively. This steadily declined to around 6.5 and 7.13 over a 4 hour period.

### ZetaSizer results

The mean particle diameter for pure IV SMOFLipid admixture and IV SMOFLipid admixture mixed with ibuprofen lysine throughout the 4 hour period was 230–250 nm (Table 5). The polydispersity index was on average 0.125 for both formulations, which indicated monodispersity. However, a small amount of particles, with approximately 4–5 μm in diameter were detected by the ZetaSizer within 2 hours of mixing.

**Table 5:** Change in mean particle diameter in IV SMOFLipid admixture and IV SMOFLipid admixture + ibuprofen combination.

Mean Particle Diameter (MD) for IV SMOFLipid admixture				
	Time = 0 hours	Time = 1 hour	Time = 2 hours	Time = 4 hours
IV SMOFLipid admixture MD (nm) ± SD	231.7 ± 1.75	232.0 ± 2.96	232.2 ± 2.52	230.7 ± 1.45
IV SMOFLipid admixture + Ibuprofen Lysine 1.25 mg/mL MD (nm) ± SD	225.4 ± 2.96	223.9 ± 2.85	228.3 ± 5.23	223.9 ± 3.76

The zeta potentials for pure IV SMOFLipid admixture and IV SMOFLipid admixture mixed with ibuprofen were around -50 to -45 mV (Table 6). This value did not change throughout the 4 hour period, suggesting both were electrically stable.

**Table 6:** Change in zeta potential in IV SMOFLipid admixture and SMOF lipid admixture + ibuprofen combination.

Zeta Potential for IV SMOFLipid admixture				
	Time = 0 hours	Time = 1 hour	Time = 2 hours	Time = 4 hours
IV SMOFLipid admixture Zeta Potential (mV) ± SD	-41.0 ± 3.38	-47.0 ± 0.69	-46.7 ± 3.84	-49.6 ± 2.45
IV SMOFLipid admixture + Ibuprofen Lysine 1.25 mg/mL Zeta Potential (mV) ± SD	-45 ± 0.63	-47.7 ± 0.68	-46.1 ± 1.10	-48 ± 1.05

### Ibuprofen recovery from TPN and LA in combination with ibuprofen

Based on the stability data from physical compatibility, Ibuprofen 1.25 mg/mL was the concentration chosen for chemical compatibility. When mixed with the starter TPN, standard preterm TPN, Low carbohydrate TPN, 10% glucose and IV SMOFLipid admixture, HPLC analysis resulted in approximately 100% recovery of drug throughout the 4 hour period (Table 7). Solvent peaks attributed to the PN or IV SMOFLipid admixture components were detected within the first two minutes of analysis.

**Table 7:** Change in ibuprofen concentration over 4 hours in ibuprofen + PN/IV SMOFLipid admixture.

Ibuprofen Lysine 1.25 mg/mL HPLC Results						
		0 hours	1 hour	2 hours	3 hours	4 hours
**Ibuprofen lysine 1.25 mg/mL + IV SMOFLipid admixture**	Sample concentration (μg/mL) ± CV	12.44 ± 6.03	12.05 ± 5.06	12.67 ± 0.16	12.83 ± 2.65	13.03 ± 3.07
	% Initial ± CV	100	96.4 ± 5.03	101.4 ± 0.18	102.6 ± 2.67	104.45 ± 3.34
**Ibuprofen lysine 1.25 mg/mL + Standard Preterm PN**	Sample concentration (μg/mL) ± CV	101.4 ± 4.2	101.9 ± 1.87	101.2 ± 3.05	101.4 ± 2.3	101.4 ± 2.4
	% Initial ± CV	100	100.6 ± 0.02	100 ± 0.07	100.2 ± 0.02	100.1 ± 0.02
**Ibuprofen lysine 1.25 mg/mL + 7.5% Dextrose Preterm PN**	Sample concentration (μg/mL) ± CV	105.8 ± 1.01	105.2 ± 1.71	107.4 ± 0.20	107.8 ± 0.70	107.0 ± 1.31
	% Initial ± CV	100	99.4 ± 0.007	101.5 ± 0.01	101.9 ± 0.02	101.2 ± 0.02
**Ibuprofen lysine 1.25 mg/mL + Starter TPN**	Sample concentration (μg/mL) ± CV	107.7 ± 1.31	105.6 ± 0.09	106.0 ± 0.87	105.2 ± 0.07	105.9 ± 0.62
	% Initial ± CV	100	98.0 ± 0.01	98.4 ± 0.02	97.7 ± 0.01	98.3 ± 0.02

## Discussion

Co-administration of drugs through IV lines is a frequent important clinical challenge because of the limited number of lines available in critically ill patients. The complex physiochemical nature of TPN and lipids makes it even more challenging. Some studies have suggested a protocol of techniques to quantitatively and qualitatively assess PN physiochemical compatibility [8–10]. This study employed some of these tests to assess visual, microscopic and chemical changes to drug-PN/lipid mixtures.

In our study, the compatibility of TPN/LA with Ibuprofen lysine was found to be concentration dependant. All TPN and Ibuprofen mixtures formed white, opaque solutions which produced a visible Tyndall Effect with ibuprofen 5 mg/mL. After 24 hours, sediment was detected at the bottom of the container, leaving the solutions clear. This observation was similar to Holt’s previous findings, which documented white precipitate [8]. Mixing ibuprofen 2.5 mg/mL with ANZNN parenteral nutrition Concensus group standard preterm and low carbohydrate preterm PN formed clear, colourless solutions free of microscopic particles. However, starter TPN did not form a clear solution with ibuprofen until the concentration was 1.25 mg/mL.

The 10% glucose formed a clear, colourless solution when mixed with ibuprofen lysine of any concentration. In addition, no drug loss was observed. This showed glucose was unlikely to be involved in the incompatibility reactions seen in the three PN formulations. These findings were consistent with a previous study that looked at ibuprofen lysine’s stability in 5% glucose solution [11]. They concluded that when protected from light, ibuprofen lysine is stable in 5% dextrose for up to 15 days.

Previous PN-drug compatibility studies have suggested pH plays an important role in determining compatibility of admixtures by affecting the solubility. Most TPN products have a pH between 5.5 and 6, which is buffered by amino acids [9, 12, 13]. This study showed pH plays a minimal role in the incompatibility reactions observed as it did not change significantly prior to and after mixing.

This was the first study that evaluated chemical compatibility of ibuprofen lysine with TPN. Over the 4 hour period, the ibuprofen lysine concentration did not change when mixed with PN or IV SMOFLipid admixture. Degradation peaks were not detected. This suggests ibuprofen lysine is not chemically reacting with any of the components in the PN or IV SMOFLipid admixture.

The baseline mean particle diameter for IV SMOFLipid admixture was between 220 to 240 nm. The polydispersity index was approximately 0.1–0.13, which suggests the emulsion is monodisperse. This was consistent with a previous report stating that SMOF particles had an average diameter of 220 to 240 nm [10]. Addition of ibuprofen lysine to the emulsion did not change the mean particle size or polydispersity index significantly over a 4 hour period. This complies with the USP recommendation that stable emulsions have particles with an average diameter of less than 500 nm [14].

## Conclusion

Ibuprofen lysine, at a concentration of 1.25 mg/mL is physically and chemically compatible with 10% glucose, starter TPN, standard preterm PN and low carbohydrate TPN formulations. IV SMOFLipid admixture combined with vitamins is chemically compatible with ibuprofen lysine 1.25 mg/mL.
